# Acute Kidney Injury Post-Percutaneous Nephrolithotomy (PNL): Prospective Outcomes from a University Teaching Hospital

**DOI:** 10.3390/jcm10071373

**Published:** 2021-03-29

**Authors:** Sunil Pillai, Akshay Kriplani, Arun Chawla, Bhaskar Somani, Akhilesh Pandey, Ravindra Prabhu, Anupam Choudhury, Shruti Pandit, Ravi Taori, Padmaraj Hegde

**Affiliations:** 1 Department of Urology, Kasturba Medical College Hospital, Manipal Academy of Higher Education (MAHE), Manipal 576104, Karnataka, India; sunil.pillai@manipal.edu (S.P.); akshaykriplani@gmail.com (A.K.); ravindra.prabhu@manipal.edu (R.P.); dranupamchoudhary86@gmail.com (A.C.); shrutirpandit0492@gmail.com (S.P.); ravitaori90@gmail.com (R.T.); padmaraj.hegde@manipal.edu (P.H.); 2 Department of Urology, University Hospital Southampton NHS Trust, Southampton SO16 6YD, UK; bhaskarsomani@yahoo.com; 3 Department of Community Medicine, Kasturba Medical College, Manipal Academy of Higher Education (MAHE), Manipal 576104, Karnataka, India; akhilesh.pandey@manipal.edu

**Keywords:** acute kidney injury, percutaneous nephrolithotomy

## Abstract

Acute Kidney Injury (AKI) after percutaneous nephrolithotomy (PNL) is a significant complication, but evidence on its incidence is bereft in the literature. The objective of this prospective observational study was to analyze the incidence of post-PNL AKI and the potential risk factors and outcomes. Demographic data collected included age, gender, body mass index (BMI), comorbidities (hypertension, diabetes mellitus), and drug history—particularly angiotensin converting enzyme inhibitors (ACE inhibitors), angiotensin II receptor blockers and beta blockers. Laboratory data included serial serum creatinine measured pre- and postoperation (12, 24, and 48 h), hemoglobin (Hb), total leucocyte count (TLC), Prothrombin time (PT), serum uric acid and urine culture. Stone factors were assessed by noncontrast computerized tomography of kidneys, ureter and bladder (NCCT KUB) and included stone burden, location and Hounsfield values. Intraoperative factors assessed were puncture site, tract size, tract number, operative time, the need for blood transfusion and stone clearance. Postoperative complications were documented using the modified Clavien–Dindo grading system and patients with postoperative AKI were followed up with serial creatinine measurements up to 1 year. Among the 509 patients analyzed, 47 (9.23%) developed postoperative AKI. Older patients, with associated hypertension and diabetes mellitus, those receiving ACE inhibitors and with lower preoperative hemoglobin and higher serum uric acid, had higher incidence of AKI. Higher stone volume and density, staghorn stones, multiple punctures and longer operative time were significantly associated with postoperative AKI. Patients with AKI had an increased length of hospital stay and 17% patients progressed to chronic kidney disease (CKD). Cut-off values for patient age (39.5 years), serum uric acid (4.05 mg/dL) and stone volume (673.06 mm^3^) were assessed by receiver operating characteristic (ROC) curve analysis. Highlighting the strong predictors of post-PNL AKI allows early identification, proper counseling and postoperative planning and management in an attempt to avoid further insult to the kidney.

## 1. Introduction

“Primum non nocere”, the preservation of renal function, is of paramount importance in the treatment of renal calculus disease, especially in view of its potential for recurrence.

Percutaneous Nephrolithotomy (PNL) is the surgical option of choice for upper urinary tract calculi with sizes of >2 cm, and selected calculi <2 cm [1]. A perceived drawback of PNL is its deleterious effect on renal function. Short- and long-term effects of PNL have been studied [2]; however, data on incidence of Acute Kidney Injury (AKI) following PNL is sparse.

Kidney Disease: Improving Global Outcomes (KDIGO) [3,4] has defined and issued practice guidelines for AKI to optimize its management [3,4,5,6,7]. AKI is diagnosed when one of the following criteria is met: an increase in serum creatinine greater than or equal to 0.3 mg/dL within 48 h; an increase in serum creatinine greater than or equal to 1.5 times baseline within the previous 7 days; urine volume less than or equal to 0.5 mL/kg/h for 6 h. Postoperative AKI is a significant complication in urology patients with an incidence rate of 6.7% to 38.2% [8], and is associated with poor postoperative outcomes, longer hospital stays, potential for requirement of intensive care and renal replacement therapy [5,6,7,8]. AKI-associated mortality has also been reported to be as high as 23% [9]. We have studied the incidence, risk factors and outcomes of post-PNL AKI. Postoperative complications were documented using the modified Clavien–Dindo grading system. Clinic review was at 1 month and patients with postoperative AKI were followed up with serial creatinine measurements for up to 1 year.

## 2. Patients and Methods

After institutional ethics committee approval and registration with the Clinical Trial registry of India (REF/2018/09/021711), we conducted a prospective observational study of consecutive patients undergoing PNL at our tertiary referral center from November 2018 to October 2019 using 4 experienced consultant endourologists. Standard PNL protocols were followed for evaluation, treatment and follow-up. Demographic data collected were age, gender, body mass index (BMI), comorbidities including hypertension, diabetes mellitus, drug history of angiotensin converting enzyme inhibitors (ACE inhibitor), angiotensin II receptor blockers and beta blockers. Laboratory data included serial serum creatinine measured pre- and postoperation (12, 24, 48 h), hemoglobin (Hb), total leucocyte count (TLC), Prothrombin time (PT), serum uric acid and urine culture. Stone factors were assessed by noncontrast computerized tomography of kidneys, ureter and bladder (NCCT KUB) and included stone burden in cubic millimeters (volume = L × W × D × π × 0.167), location and Hounsfield values and laterality. The intraoperative factors assessed were puncture site, tract size, tract number, operative time, the need for blood transfusion, stone clearance, usage of ureteral stent or nephrostomy tube and any ancillary procedures.

The operative procedure followed a standardized prone PNL protocol under general anesthesia and intravenous 3rd generation cephalosporin at induction. A sterile preoperative urine culture was ensured in all patients. All patients underwent preliminary cystoscopic insertion of a 5/6Fr ureteral catheter. Dilatation after initial puncture was carried out using serial metallic Alken dilators for conventional PNL (>24Fr) and a single-step metal dilator for the miniaturized PNL (<22Fr). The commonest tract size was 28Fr (34.8%). The irrigation fluids used during percutaneous surgery were prewarmed to body temperature in our operating room and were gravity-assisted only, with no manual pressure irrigation. Pneumatic lithotripsy, using Swiss lithoclast, was carried out for all the conventional PNLs. LASER fragmentation using a 365 μm fiber was carried out in the miniaturized PNL group. All patients had a DJ stent indwelled. Operative time was calculated from the initial puncture to final skin suture.

Postoperative blood parameters included Hb, TLC, and serum creatinine at 12, 24 and 48 h as per the clinical condition. All routine blood samples were taken at 06:00 hours. No specific diet was recommended in the immediate postoperative period. Adequate hydration was advised to all patients to maintain clear urine. Analgesia was provided using parenteral tramadol. Postoperative complications were documented using the modified Clavien–Dindo grading system [10]. This is depicted in Table 1. Patients with up to Grade 1 complications were discharged on postoperative day 2. Clinical review was at 1 month and patients with postoperative AKI were followed up with serial creatinine values up to 1 year. AKI was defined as an increase in serum creatinine ≥0.3 mg/dL within 48 h. Chronic Kidney Disease (CKD) was defined by an estimated glomerular filtration rate (eGFR) of <60 mL/min/1.73 m^2^. eGFR was calculated by using the four variable Modification of Diet in Renal Disease (MDRD) formula.

Statistical analysis was carried out on SPSS, Version 16.0. Categorical variables are expressed in frequencies with percentages and were compared using Chi-square or Fisher’s exact test. Continuous variables with normal distributions are expressed as mean and standard deviation and were compared using Student’s *t*-test; those with skewed distributions are expressed as medians and interquartile range with comparison using Mann–Whitney test; a *p*-value ≤ 0.05 was considered significant. Univariate analysis was carried out to assess the relation between the dependent variable (occurrence of AKI) and each of the independent variables. Multivariate analysis was then performed using logistic regression to establish the predictive factors for the development of AKI. A receiver operating characteristic (ROC) curve was constructed, and a value of area under the curve above 0.65 was considered a cut-off value for the variable.

## 3. Results

**Figure 1:**
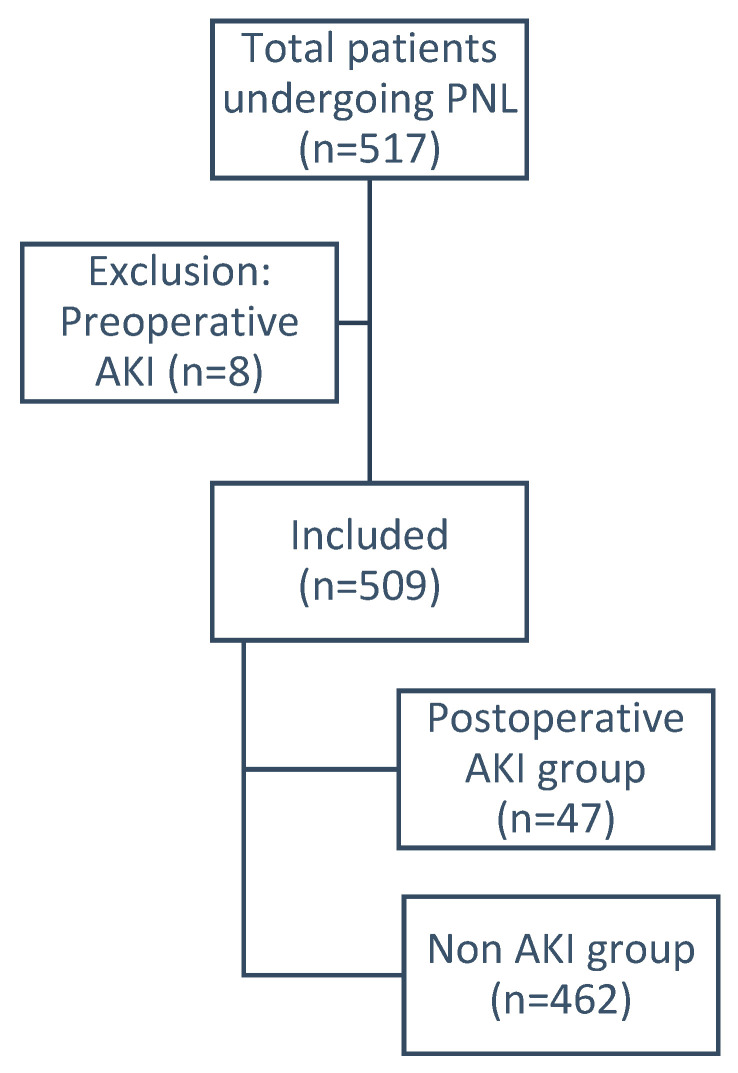
Flow chart of patients during the study period. AKI-Acute Kidney Injury; PNL-Percutaneous Nephrolithotomy.

Of 517 patients, 8 (1.5%) who had preoperative AKI were excluded (Figure 1). There were no patients with a solitary kidney in this study. All patients had normal contralateral kidneys. Three patients had a history of previous PNL in the ipsilateral unit, and one patient had history of PNL in the contralateral unit. No other renal procedures were noted in other patients. Of the remaining 509, the mean age was 48.1 ± 13.92, with 388 (76.2%) males and 121 (23.8%) females. Ninety-four (18.5%) and 142 (27.9%) patients had diabetes Mellitus and hypertension, respectively, and 47 (9.23%) developed postoperative AKI.

Details of patient demographics and stone characteristics with the univariate analysis for independent predictive factors for development of postoperative AKI are mentioned in Table 2, Table 3 and Table 4. Those with AKI were older (mean age 54.8 ± 13.9 vs. 47.4 ± 13.7 years, OR = 1.041, 95% CI = 1.017–1.066, *p* = 0.001), significantly more likely to have hypertension (51.1% vs. 25.5%, OR = 3.042, 95% CI = 1.655–5.593, *p* = 0.0002), diabetes mellitus (29.8% vs. 17.3%, OR = 2.026, 95% CI = 1.037–3.959, *p* = 0.036), have received ACE inhibitors (10.6% vs. 3.7%, OR = 3.116, 95% CI = 1.095–8.871, *p* = 0.036), have lower preoperative hemoglobin (12.6 ± 2.25 vs. 13.3 ± 1.86, *p* = 0.013) and have higher serum uric acid (5.2 ± 1.46 vs. 3.9 ± 1.44, OR = 1.758, 95% CI = 1.336–2.315, *p* = 0.00001) as compared to those without AKI. Stone volume (mm^3^) (2117.9 (761–12,452) vs. 825 (503–1573) *p* = 0.0000001), stone density (817.4 ± 439.76 vs. 985.2 ± 253.98, *p* = 0.0001) and number of staghorn stones (12.8% vs. 3.2%, OR = 4.361, 95% CI = 1.605–11.846, *p* = 0.008) were significant higher in those with AKI.

Among operative characteristics (Table 3), those with AKI had a significantly greater number of punctures (8.5% vs. 1.7%, OR = 5.279, 95% CI = 1.527–18.248, *p* = 0.019) and longer operative time (63.5 ± 21.8 vs. 55.2 ± 15.9 min, OR = 1.028, 95% CI = 0.983–1.049, *p* = 0.001). Forty-five patients in the AKI group had complete stone clearance with a stone free rate (SFR) of 95.7%. None of our patients had persistent intraoperative or postoperative hypotension requiring inotropic support. In total, two patients underwent selective angioembolization in our study.

Multivariable logistic regression analysis further demonstrated that factors significantly associated with postoperative AKI were gender (male, OR = 0.129, 95% CI = 0.021–0.787, *p* = 0.026), BMI (OR = 0.712, 95% CI = 0.550–0.923, *p* = 0.010), use of ACE inhibitors (OR = 60.404, 95% CI = 1.619–2253.49, *p* = 0.026) serum uric acid (OR = 2.163, 95% CI = 1.459–3.209, *p* = 0.0001) and puncture site (OR = 0.054, 95% CI = 0.003–1.121, *p* = 0.059). Prothrombin time and tract size were found to not be statistically significant in the preliminary analysis and were excluded from the subsequent univariate and multivariate analyses. All other variables were included.

The ROC curve was built for the variables, including age, serum uric acid and stone volume, to better define the independent predictive ability of the variables that were clinically and statistically important in both the univariate and multivariate analyses. ROC analysis was carried out to generate a cut-off value that would be informative for urologists to decide on intensive care unit (ICU) requirement and prognosis. In the ROC analysis, patients with ages greater than 39.5 years had 81% sensitivity and 26.9% specificity; those with serum uric acid levels greater than 4.05 mg/dL had 90.1% sensitivity and 55.2% specificity, with an area under curve of 79.1%; those with stone volume greater than 673.06 mm^3^ had 90.5% sensitivity and 46.3% specificity and area under curve of 70.7%; these were all associated with development of AKI. Three (6.38%) patients required postoperative hemodialysis in view of oliguria and hyperkalemia. Two of these patients required two sessions for clinical recovery, whereas the third patient recovered after a single session. Among the AKI cohort, the mean creatinine values preoperation, immediately postoperation, at the time of discharge and at the one-year follow-up were 1.3 ± 0.766, 1.3 ± 0.99, 5.05 ± 22.01 and 1.7 ± 1.12, respectively. Serum creatinine was significantly higher by 0.249 mg/dL (*p* = 0.010, 95% CI = 0.063–0.435) at one year as compared to postoperative values and eight patients (17.02%) in the AKI group progressed to CKD at the 1 year follow-up.

## 4. Discussion

Renal function can be affected by stone disease or obstruction related to it, urinary infections and by surgical intervention. Though the intent of treatment is to improve renal function, it is plausible that it could have an adverse effect. The risk of impairment exists for all levels of invasiveness—from SWL (elvi-ureteric junction.) to URS (Ureterorenoscopy) and PNL (Percutaneous Nephrolithotomy). The choice of treatment depends on stone factors, patient factors including comorbidities, surgical expertise, and also underlying renal function. A systematic review by Reeves et al. suggests that the overall renal function is not always detrimentally affected by endourological interventions [2]. Morbidities after PNL such as fever, bleeding, pleural or visceral injury and significant nephron loss have been well described [11]. Incidence of postoperative AKI for major open urological procedures varies from 6.7% to 38.2% [5,6,7,8,9]. Surprisingly, very few studies report complications of PNL and AKI [12,13,14]. This may be because impairment of renal function in the absence of significant perioperative complications appears to be minimal, transient and focal. Effect on renal function is influenced by preoperative renal status and presence of comorbidities such as diabetes mellitus and hypertension [15]. Violation of the renal parenchyma, high irrigation pressure, tract multiplicity, preoperative urine infection and postoperative bleeding are reported as attributes causing post-PNL AKI [2,16]. However, subsequent improvement in renal function is seen in almost all renal units that are obstructed and infected [15,17]. Standardized definition of AKI was introduced to aid early detection and management and improve the overall patient outcomes [3]. As AKI may be associated with mortality in up to 23% patients [9], with increased duration of hospital stay and requirement of intensive care, every attempt should be made to identify predisposing factors and predictors of postoperative AKI. 

In our prospective study, we found an incidence of post-PNL AKI of 9.2%, which was comparable to incidence reported in the literature [5,6,7,8,9]. We divided the AKI predictors into patient factors, stone factors and operative factors. Patient factors associated with a higher risk for AKI were older age, presence of comorbidities such as hypertension and diabetes mellitus, and preoperative use of ACE inhibitors or angiotensin II inhibitors, which may be due to loss of renal reserve or decreased glomerular filtration rate (GFR) due to these factors. 

Reduced plasma volume, cardiac and neuronal changes, leading to intraoperative hypotension in elderly patients, are described as causing postoperative AKI [13,18]. Autonomic neuropathy due to diabetes is known to cause perioperative hemodynamic changes [19]. Persistent hypotension in the postoperative period leads to deterioration of renal function. None of our patients had persistent intraoperative or postoperative hypotension requiring inotropic support. Alteration in the renin angiotensin system due to use of ACE inhibitors or angiotensin II inhibitors is a known predisposing factor for renal hypoperfusion, as was seen in 10.6% of patients with AKI in our cohort, making these drugs an independent predictor of AKI [20]. Low hemoglobin and leukocytosis were predictive of post-PNL AKI in our study. 

The lack of evidence in the literature makes it difficult to explain the correlation of low hemoglobin with postoperative AKI, but infection related leukocytosis may affect AKI by affecting inflammatory mediators in microcirculation. High serum uric acid levels are another risk factor for AKI, in agreement with other reported studies [21]. Crystal-independent mechanisms and crystal-dependent pathways are postulated for this. High serum uric acid can induce renal vasoconstriction and impair autoregulation, which results in reduced renal blood flow and GFR. The proposed mechanism responsible is the activation of proinflammatory cascade leading to endothelial dysfunction, which causes impaired autoregulation and renal vasoconstriction [21,22,23]. High serum uric acid levels could therefore be potentially used to help identify patients at high risk of developing AKI [21].

Stone factors such a high stone volume, density and staghorn calculi increases the complexity of the procedure and operative time, with increased risk of perioperative bleeding and infective complications, leading to AKI [14,23]. These may serve as surrogate markers for development of AKI as also observed in our study. 

Literature evidence suggests multiple tracts and larger tract size causes significant nephron damage and leads to AKI [14,24,25,26]. However, we did not find tract size to be an independent significant factor in this study. Though no morphological or functional decline by imaging and nuclear studies at the access site has been studied in the literature, it can be interpreted that the presence of multiple tracts and larger tracts cause cellular injury. Emerging urinary biomarkers such as neutrophil gelatinase-associated lipocalin (NGAL), predictive of ischemic AKI or AKI in transplant kidney after renal biopsy, have been reported in the literature [27]. In our study, cut-off values of age (39.5 years), serum uric acid (4.05 mg/dL) and stone volume (673.06 mm^3^) showed high sensitivity to predict postoperative AKI.

AKI commonly leads to increased length of hospital stay [12,28]. In our study, the mean length of hospitalization was not increased in the AKI group due to a lack of clinical deterioration in this cohort of patients. The majority were therefore managed conservatively, while 6.38% patients required renal replacement therapy. However, progression to CKD can be a sequelae of AKI [29], although complete improvement in renal function after 6–12 months has also been reported [14,30,31]. In our study, 17.02% patients in the AKI cohort progressed to CKD.

A large sample size and medium-term follow-up provided strength to this study, highlighting the strong predictors of post-PNL AKI. Counseling and postoperative planning in consultation with nephrology, avoidance of nephrotoxic drugs and appropriate fluid management are key to avoiding further insult to the kidney. Lack of a control group testing against other interventions and a urolithiasis scoring system for percutaneous nephrolithotomy outcomes, such as the Guy’s scoring system, are limitations of our study.

## 5. Conclusions

Up to 10% patients can develop post-PNL AKI, of which one-fifth can progress to CKD. Older age, presence of hypertension, diabetes mellitus, low hemoglobin, leukocytosis, high uric acid levels, staghorn calculi, use of multiple tracts and longer operative times all predict the development of AKI. Highlighting the strong predictors of post-PNL AKI allows early identification, proper counseling and postoperative planning in an attempt to avoid further insult to the kidney and care must be taken to optimize these conditions to minimize AKI.

## Figures and Tables

**Table 1:** Clavien–Dindo complications after percutaneous nephrolithotomy (PNL): *n* = 517.

Complication	Clavien–Dindo	15Fr	22Fr	24Fr	26Fr	28Fr	30Fr	32Fr	34Fr	36Fr	*p*-Value
Fever	2	8	0	3	8	18	4	12	1	2	0.743
Haematuria	1	0	0	2	5	3	1	8	0	0	0.342
Angioembolization	3B	0	0	1	3	2	0	0	0	0	0.663
Auxiliary proc.											0.143
URS		0	0	0	2	3	1	1	0	0	
2nd PNL		0	0	0	2	0	0	2	0	0	
Bladder wash		0	0	1	2	0	0	2	0	0	
Stent reposition	3A	0	0	0	0	0	1	0	0	0	
Visual internal Urethrotomy		0	0	0	0	0	0	0	0	1	

URS: Ureterorenoscopy.

**Table 2:** Patient characteristics, preoperative laboratory values and stone characteristics.

Variables		All Patients (*n* = 509)	AKI Cohort (*n* = 47)	Non-AKI (*n* = 462)	*p*-Value
**Patient Characteristics**					
Age (years) (mean ± SD)		48.13 ± 13.92	54.83 ± 13.907	47.45 ± 13.75	0.001
Gender (M)		388 (76.2%)	39 (83%)	349 (75.5%)	0.254
Gender (F)		121 (23.8%)	8 (17%)	113 (24.5%)	
BMI (kg/m^2^)		25.23 ± 2.94	25.21 ± 3.12	25.23 ± 2.92	0.974
Hypertension		142 (27.9%)	24 (51.1%)	118 (25.5%)	0.0002
Diabetes mellitus		94 (18.5%)	14 (29.8%)	80 (17.3%)	0.036
ACE inhibitors		22 (4.3%)	5 (10.6%)	17 (3.7%)	0.043
Beta-blockers		10 (2%)	1 (2.1%)	9 (1.9%)	1.00
**Preoperative Laboratory Values**					
Hemoglobin (mg/dL)		13.29 ± 1.91	12.63 ± 2.25	13.36 ± 1.86	0.013
Platelet(/µL)		273,669.36 ± 79,821.98	276,833.33 ± 103,392.68	273,354 ± 77,278.68	0.778
Prothrombin time (s)		10.58 ± 0.39	10.75 ± 0.66	10.55 ± 0.32	0.006
Creatinine (mg/dL)		1.42 ± 4.30	1.34 ± 0.76	1.43 ± 4.5	0.895
Uric Acid (mg/dL)		4.13 ± 1.52	5.23 ± 1.46	3.91 ± 1.44	0.00001
Total leucocyte count (/mm^3^)		8.73 ± 3.84	9.73 ± 9.54	8.63 ± 2.65	0.06
**Stone Characteristics**					
Stone Volume (mm^3^) (median (Q1–Q3))		880.95 (524.38–1801.25)	2117.94 (761–12,452)	825 (503–1573)	0.00
Hounsfield Unit (HU)		970.59 ± 278.55	817.45 ± 439.76	985.18 ± 253.98	0.0001
Stone location	Upper Calyx	26 (5.1%)	2 (4.3%)	24 (5.2%)	1.000
	Middle Calyx	53 (10.4%)	9 (19.1%)	44 (9.5%)	0.074
	Lower Calyx	138 (27.1%)	15 (31.9%)	123 (26.6%)	0.437
	Pelvic	190 (37.3%)	14 (29.8%)	176 (38.1%)	0.262
	PUJ	153 (30.1%)	12 (25.5%)	141 (30.5%)	0.477
	Staghorn	21 (4.12%)	6 (12.8%)	15 (3.24%)	0.008

ACE, Angiotensin converting enzyme; PUJ, Pelvi-ureteric junction.

**Table 3:** Intraoperative data.

Variables		All Patients (*n* = 509)	AKI Cohort (*n* = 47)	Non-AKI (*n* = 462)	*p*
Puncture site	Supracostal	75 (14.7%)	5 (10.6%)	70 (15.2%)	0.406
	Infracostal	434 (85.3%)	42 (89.4%)	392 (84.8%)	
Tract size (Fr) (median (Q1–Q3))		28 (26–32)	28 (26–28)	28 (26–32)	0.032
Puncture Number	Single Puncture	497 (97.6%)	43 (91.5%)	454 (98.3%)	0.019
	>1 Puncture	12 (2.35%)	4 (8.51%)	8 (1.73%)	
Blood Transfusion		15 (2.9%)	3 (6.4%)	12 (2.6%)	0.153
Operative time (minutes)		55.99 ± 16.71	63.51 ± 21.79	55.23 ± 15.93	0.001

**Table 4:** Univariate and multivariate logistic regression analyses for predictors of post-PNL Acute Kidney Injury (AKI).

Variable		Univariate Analysis		Multivariate Analysis	
		Unadjusted OR	*p*-Value	Adjusted OR	*p*-Value
Age		1.041 (1.017–1.066)	0.001	1.050 (0.998–1.105)	0.060
Gender	Male	1.578 (0.717–3.477)	0.257	0.129 (0.021–0.787)	0.026
	Female	1.0		1.0	
BMI		0.998 (0.901–1.106)	0.974	0.712 (0.550–0.923)	0.010
Hypertension	Yes	3.042 (1.655–5.593)	0.0003	2.514 (0.699–9.035)	0.158
	No	1.0		1.0	
Diabetes Mellitus	Yes	2.026 (1.037–3.959)	0.039	2.423 (0.521–11.260)	0.259
	No	1.0		1.0	
ACE inhibitors	Yes	3.116 (1.095–8.871)	0.033	60.404 (1.619–2253.49)	0.026
	No	1.0		1.0	
Beta-blocker	Yes	1.094 (0.136–8.830)	0.933	0.770 (0.031–19.033)	0.873
	No	1.0		1.0	
Creatinine		0.994 (0.911–1.085)	0.896	1.332 (0.861–2.059)	0.198
Uric Acid		1.758 (1.336–2.315)	0.00005	2.163 (1.459–3.209)	0.0001
Total leucocyte count		1.045 (0.989–1.103)	0.116	0.999 (0.841–1.187)	0.988
Operative Time		1.028 (0.983–1.049)	0.001	1.015 (0.982–1.049)	0.364
Blood Transfusion (*n*)	Yes	2.557 (0.695–9.405)	0.158	8.408 (0.396–178.42)	0.172
	No	1.0		1.0	
Stone size		1.000		1.000	
Stone Location (*n*)	Upper calyx	0.811 (0.186–3.545)	0.781	0.223 (0.011–4.509)	0.328
	Middle calyx	2.250 (1.021–4.959)	0.044	1.822 (0.269–12.370)	0.539
	Lower calyx	1.292 (0.676–2.467)	0.438	1.843 (0.336–10.121)	0.482
	Pelvis	0.689 (0.359–1.324)	0.264	1.897 (0.333–10.796)	0.471
	PUJ	0.478 (0.394–1.548)	0.781	1.582 (0.205–12.207)	0.660
	Staghorn	4.361 (1.605–11.846)	0.004	0.594 (0.032–10.944)	0.726
Tract number (*n*)	Single Tract	1.000		1.000	
	>1 Tracts	5.279 (1.527–18.248)	0.009	89.698 (0.795–10,119.9)	0.062
Tract site (*n*)	Supracostal	0.667 (0.255–1.744)	0.408	0.054 (0.003–1.121)	0.05
	Infracostal				

(OR—Odds Ratio).

## Data Availability

NA.
